# Comparison of treatments for bullous keratopathy in rabbits

**DOI:** 10.3892/etm.2013.1025

**Published:** 2013-03-21

**Authors:** HAIXIA ZHAO, YUNNA LUO, CHUNMEI NIU, WENYING GUAN

**Affiliations:** Department of Ophthalmology, The Affiliated Hospital of Inner Mongolia Medical College, Hohhot, Inner Mongolia 010050, P.R. China

**Keywords:** bullous keratopathy, deep lamellar endothelial keratoplasty, keratoplasty, penetrating, rabbits

## Abstract

The aim of the present study was to compare deep lamellar endothelial keratoplasty (DLEK) and penetrating keratoplasty (PK) treatments for bullous keratopathy (BK). In total, 36 healthy New Zealand white rabbits were randomly divided into 3 groups termed the experimental, DLEK and PK groups. The experimental control group received no treatment. The DLEK and PK groups were observed for corneal astigmatism at 1, 2, or 3 months post-surgery using a corneal topography instrument and a slit lamp microscope. The incidence of immune rejection after 3 months of recovery was determined using hematoxylin and eosin (H&E) staining. The corneal specimens from the surgery groups were compared with those from the control group. In the 12 rabbit eyes that underwent the DLEK surgery, the central cornea became clear after 1 week. After 3 months, these corneas were almost transparent and no eye infections or other complications were observed in 10 of the eyes, while surgical perforations in 2 eyes led to surgical lamellar failure. In the PK surgery group, in which 12 rabbit eyes were also treated, nine were almost transparent after 3 months of recovery, while three eyes were immunologically rejected due to the corneal grafts. The occur-rences of corneal astigmatism that were observed following DLEK and PK treatment were significantly different after 1, 2 and 3 months of recovery (P<0.05). Normal corneal staining was observed in the DLEK and PK rabbits subjected to H&E staining after 3 months of recovery. A BK animal model was established by curetting the Descemet’s membrane (DM film). In comparison with PK, DLEK is a superior surgical treatment for BK.

## Introduction

Bullous keratopathy (BK) develops as a result of corneal endothelial cell damage or a reduction in the number of corneal endothelial cells due to lesions. Corneal endothelial cell dysfunction is often a late clinical development. Intraocular lens implantation has become popular and widespread due to the application of laser technology and the increased incidence of BK, which cannot be cured using drugs or conservative treatments (1–5). For a number of years, penetrating keratoplasty (PK) has been the standard treatment for BK (6). Although the success rate of this procedure is as high as 90%, the post-operative complications are a serious clinical problem. During the 20th century, high precision surgical instruments and microscopes, medicines, anti-immune rejection medicines and improvements in corneal surgery, as well as corneal topography techniques, have been developed. Archila *et al*(7) attempted the first clinical implementation of a corneal flap following lamellar keratoplasty. Since the publication of the study, optical corneal transplantation has been widely performed in China (8). Clinicians have made advancements in elements of the cornea transplant procedure and corneal transplantation has become accurate to the corneal cell level (9). Deep lamellar endothelial keratoplasty (DLEK) is the type of lamellar keratoplasty (10,11) that is most often used for the treatment of corneal endothelial cell lesions. Lamellar corneal transplantation is often used to specifically address these lesions.

In the current study, a 1-mm syringe needle was inserted into the anterior chamber of the cornea and the Descemet’s membrane (DM film) and endothelial cells were removed to create the rabbit BK model. The results of PK and DLEK surgeries were assessed after 1, 2 and 3 months of recovery by observing the corneal topography and morphology and the presence or absence of an immune rejection and corneal astigmatism. The aim of the present study was to identify a safe and effective surgical treatment for BK and to support the validity of this approach using experimental findings (12–14).

## Materials and methods

### Experimental animals

A total of 36 healthy and eye disease-free New Zealand white rabbits (male and female; Inner Mongolia Medical College Hospital Animal Experimental Center, Hohhot, China), weighing 1.5–2.0 kg, were maintained at room temperature.

The 36 rabbits were randomly divided into the DLEK, PK and experimental control group. Each group had 12 rabbits. The DLEK and PK groups were further divided into groups that were observed 1, 2 or 3 months subsequent to the surgery. The right eye of each rabbit was used as the experimental eye (for surgery). The corneal donor group (12 rabbits, 24 eyes) provided corneas to the DLEK and PK graft groups. This study was carried out in strict accordance with the recommendations in the Guide for the Care and Use of Laboratory Animals of the National Institutes of Health. The animal use protocol has been reviewed and approved by the Institutional Animal Care and Use Committee (IACUC) of the Affiliated Hospital of Inner Mongolia Medical College.

### Experimental models

SU-MIAN-XIN (0.2 ml/kg), made by the Veterinary Research Institute of the Military Medical Science Academy of the PLA in Beijing, China, was administered to the rabbits and then the animals were anesthetized with 0.5% tetracaine. Each rabbit was covered with a surgical drape and its eyelid was opened. Using a microscope for visualization, a surgical knife was used to puncture the cornea 1 mm from the limbus. The wound was closed and a 1-mm syringe needle was inserted into the anterior chamber of the cornea and bent at an obtuse angle. The DM film was curetted and the anterior chamber was washed with BSS to establish the characteristics of BK. Each animal was observed using a slit lamp following the surgery and examined every 4 weeks to test for corneal opacity, corneal edema, thickening of the cornea that exceeded >3 times its normal thickness, hyphema, ocular infections, glaucoma or other complications. The rabbit models of BK were confirmed to be steadied prior to determining that the animals were ready for surgical treatment.

### Pre-operative preparation

Prior to surgery, the conjunctival sac of the right eye was treated with 1% pilocarpine eye drops (to induce miosis) and a gentamicin and saline flush. Each animal was placed in the left lateral position while under general anesthesia. Next, the animal’s eyelid was disinfected, the area was draped and a speculum was used to open the eyelid.

### DLEK group

Twelve eyes were obtained from the corneal donor group to construct a donor cornea transplant sample of DLEK. The corneal epithelium was removed by swabbing it with a sponge while the animal’s cornea was visualized using a microscope. A micro knife was used to make a corneal lamellar incision that was 120–160 *μ*m thick, with a diameter of 9.0–9.5 mm and a pedicle flap. The flap was removed; following the lamellar corneal cutting procedure, a ring with a diameter of 7.25 mm was used to confirm the complete separation of the grafts and the removal of the original tissue.

The right eyes from the DLEK group were used to construct a receptor cornea transplant bed of DLEK. After using a swab to wipe the corneal epithelium that was visualized using a microscope, a corneal lamellar knife was used to make an incision 120–160 *μ*m thick with a diameter of 9.0–9.5 mm. The corneal flap was removed. Using a 7.0-mm trephine drill to remove part of the corneal tissue for receptor cornea planting tablet of DLEK. The corneal tissue was removed which in addition to the recipient rabbit’s corneal stroma, which included the nether lamellar layer, Descemet’s layer and the corneal cortex. An incision was made in the cornea to a depth of two-thirds of its thickness. The structure was completely separated from the planting bed using a drill subsequent to the clamp clip being removed.

The corneal transplant process of DLEK was conducted as follows: The donor corneal endothelium was placed face down on the corneal bed and the graft was fixed using interrupted sutures. The pedicle flap of the corneal tissue was reset and closed using combined, interrupted sutures.

### PK group

Twelve eyes from corneal donor groups were used to create transplant samples of PK. Using a microscope to visualize the cornea, a cut was made using a drill with a diameter of 7.25 mm. Drilling was stopped when a loss of resistance was felt by the operator. The complete separation of the grafts was then confirmed. Care was taken to ensure that the grafts did not move and were kept in reserve.

The right eyes of the PK group were used to create receptor cornea transplant bed and corneal transplant process of PK. The corneal beds were prepared for the transplant using the same method of graft production that was described earlier. A hole 7.0 mm in diameter was drilled in the cornea and the corneal pieces were removed to complete the preparation of the bed.

The donor corneal endothelium was placed face down on the graft and then attached to the DM using interrupted sutures.

### Post-operative treatment

The rabbits were handled using routine post-operative care. The sutures were removed as required after 1 month of recovery. The rabbits’ eyes were examined after 3 months of recovery. The animals were euthanized and the eyes were removed. For each eye, the cornea was separated from the limbus and stored in fixative as preparation for hematoxylin and eosin (H&E) staining.

### Post-operative observation

The eyes were observed once a week using slit lamp microscopy and assessed for the presence of new blood vessels in the corneal bed, graft edema, opacity, melting, slab gap, epithelial growth on the transplant, the presence of immune rejection and any morphological changes of the cornea. The topography was examined after 1, 2 and 3 months of recovery and any changes in the astigmatism (Astig) values were recorded. Subsequent to 3 months of recovery, two rabbits from the PK group and two rabbits from the DLEK group were chosen for corneal H&E staining to observe the cornea-receptor interface.

### Statistical analysis

The data were analyzed using SPSS 13.0 software for comparison of the groups and are expressed as mean ± standard deviation (SD). A t-test was used for a single factor analysis to compare the experimental and control groups. P<0.05 was considered to indicate a statistically significant difference.

## Results

### DLEK group

At day 1 post-surgery, 10 of the rabbits’ eyes exhibited severe eyelid spasms, moderate conjunctival hyperemia, corneal opacity (grade I) and moderate edema. After 2 weeks, the conjunctival congestion was alleviated and the cornea became clear in the center, although the periphery remained pale and opaque. The corneal graft remained in place and the graft edema persisted (grade I). The sutures were intermittently removed after 1 month and the blepharospasms were significantly reduced at this time. The cornea gradually became transparent, although the periphery remained white and opaque. The graft edema disappeared and a small amount of corneal neovascularization was observed. After 3 months of recovery, it was possible to flip the eyelid and the rabbits remained emotionally stable. The central section of the transparent cornea became clear, although the peripheral cornea remained off-white. No eye infections or other complications were observed. In the DLEK group, 2 cases of perforated corneal flaps occurred which caused the surgery to be terminated.

### PK group

In the PK group, 9 of the 12 rabbit eyes exhibited severe eyelid spasms at day 1 post-surgery. The rabbits were confused and moderate conjunctival hyperemia, corneal edema (grade II) and turbidity (grade II) were observed. After day 7, the corneal edema persisted, the periphery was a hazy gray, the corneal sutures remained in place and numerous new blood vessels (with a length of 1–2 mm) were observed between the corneal limbus and cornea. At ∼2 weeks post-surgery, the conjunctival hyperemia was reduced and the central section of the cornea became transparent. The area surrounding the grafts was a hazy gray and the corneal limbal neovascularization grew to between 2 and 3 mm. The corneal sutures were removed after 1 month of recovery. The corneal neovascularization subsided and no endophthalmitis was observed. At ∼3 months post-surgery, the rabbits remained emotionally stable when the eyelid was flipped and the cornea was nearly transparent. At this time, 3 eyes exhibited severe corneal opacity and numerous new blood vessels covered the entire graft. Corneal melting (grade I) was observed and an immune graft rejection occurred.

### Corneal Astig value

**Figure 1:**
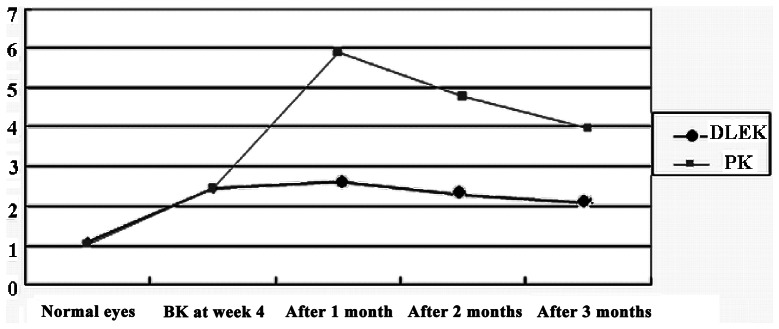
Pre- and post-operative Astig values for rabbits undergoing DLEK or PK corneal surgery. Astig, astigmatism; DLEK, deep lamellar endothelial keratoplasty; PK, penetrating keratoplasty.

In the DLEK group, the post-operative corneal Astig value was significantly different from that of the normal rabbit eyes (P<0.05) at several time points. Compared to the BK 4 week, the mean corneal Astig value was signifcantly decreased at 3 months after surgery (t=10.251, P<0.05, n=10). Compared to the observed normal rabbit eyes at several time points, the mean corneal Astig value was signifcantly increased in the PK group (P<0.05). At 3 months subsequent to the corneal surgery, the Astig value was significantly different from the BK 4 week value (t= −78.184, P<0.05, n=10). Compared to the PK group at several time points after surgery, the corneal Astig value was also signifcantly decreased in the DLEK group (F=3833.614, P<0.05; Table I and Fig. 1).

### Histological changes

**Figure 2:**
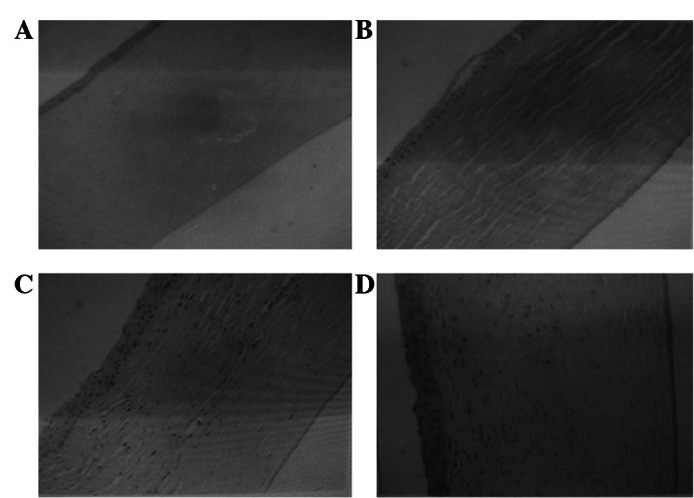
(A) A normal cornea (H&E, magnification, ×200); (B) a normal corneal endothelium attached to the DM membrane of endothelial cells following simple curettage at 4 weeks (H&E, magnification, ×100); (C) a cornea 3 months after DLEK surgery (H&E, magnification, ×100); (D) a cornea 3 months after PK surgery (H&E, magnification ×100). H&E, hematoxylin and eosin; DM, Descemet’s membrane; DLEK, deep lamellar endothelial keratoplasty; PK, penetrating keratoplasty.

Normal corneal tissue has 4 layers; the epithelium, stroma, DM and the endothelial cell layer membrane (Fig. 2A). In 2 rabbits, the DM appeared as a layer of endothelial cells attached to the membrane at 4 week post-surgery (Fig. 2B). The continuity of the collagen fiber interface was established in the DLEK group subsequent to 3 months of recovery (Fig. 2C), and the corneas in the PK group were essentially normal at this time (Fig. 2D).

## Discussion

Corneas have been used for tissue and organ transplants for 100 years and are the most commonly transplanted organ. Many patients with corneal blindness are able to sense light following this type of transplant. The aim of a corneal transplant is to replace an opaque cornea with healthy corneal tissue and to restore a patient’s sight. Each year, 3,000 corneal transplants are conducted in China, 40,000 in the United States and 2,000 in Japan (15).

Future experimental studies using DLEK for the treatment of BK require the establishment of a reliable animal model. Rabbits are the most commonly used animal model in ophthalmology studies (17–19), however, the rabbit and human corneal endothelia differ in their biological characteristics, mainly in terms of endothelial regeneration (16–18). The establishment of a stable and lasting corneal endothelial injury model is worthwhile. The results of the present study suggest that scraping the endothelium and the basement membrane of corneal endothelial adhesions (the DM film) may significantly delay the regeneration of rabbit corneal endothelial cells and prolong the effects of endothelial cell injury. These effects are in agreement with the clinical pathological characteristics of BK and support the use of this animal model for evaluating the effects of an experimental DLEK surgery accurately and objectively.

Langerhans cells within the corneal epithelium express HLA antigens, which give these cells the ability to recognize non-self antigens. Compared with PK, in theory, DLEK is able to decrease the risk of antigenic material production as the graft does not contain epithelial cells. As the donor epithelial tissue has been separated from the patient’s own corneal stroma, the Langerhans cells are not in direct contact with donor antigens. Consequently, the incidence of graft rejection is reduced following this technique compared with that in the matrix model. However, immune-mediated corneal graft rejection is mainly caused by an endothelial rejection, as the endothelial cells may express allogeneic antigens which may cause an endothelial rejection. In the present study, during observations of the two groups, several rabbits from each group developed an immune rejection. In the DLEK group, a small amount of corneal neovascularization appeared after 1 month and had increased by 3 months post-surgery. When the corneal sutures were removed, the corneal neovascularization gradually subsided and the central part of the cornea became transparent. The majority of the corneal neovascularization grew ∼1–2 mm into the limbus area in the PK group and by the second week following surgery, the corneal limbal neovascularization had increased to 2–3 mm into the limbus area. At ∼3 months after this increase, three corneas were still cloudy with numerous new blood vessels and they appeared to be undergoing a severe immune graft rejection. In the present study, the immune rejections were more severe and occurred more frequently in the PK group than in the DLEK group. Corneal allograft rejection is a trigger mechanism that may be a complex process affected by numerous factors. The following factors were identified using a multi-factor analysis of this process: i) conducting thick graft surgery (which results in excessive antigen release from the allogeneic tissue); ii) stimulation by the corneal sutures causes neovascularization, which may lead to an immune graft rejection (a large diameter graft is more susceptible to corneal neovascularization than smaller grafts); iii) a larger number of Langerhans cells, which are distributed in the peripheral cornea, possess antigen-presenting functions and are stimulated by various physical and chemical factors. These factors may all increase the number of Langerhans cells, which gradually migrate to the central corneal graft position or deviate from the central cornea to the periphery. All of these factors may lead to rejection.

PK completely replaces the diseased cornea with allogeneic tissue. In an ocular surface reconstruction, the corneal thickness and curvature of the recipient’s original cornea are not identical to those of the donor cornea. In addition, suturing in several directions and with several suture tensions may distort the corneal surface, leading to a highly irregular post-operative astigmatism. DLEK retains the anterior corneal surface, the cortex, the first elastic layer and a portion of the matrix layer of the recipient, therefore, the anterior corneal surface remains smooth and the astigmatic curvature of the cornea that is caused by inconsistencies and sutures is reduced. Consequently, DLEK causes less astigmatism than PK. An analysis of the causes of astigmatism may prevent the formation of highly irregular astigmatism. In the present study, the corneal topography astigmatism values in the pre-operative PK group, the BK group at 4 weeks and at several post-operative time points were significantly different when compared with those of the DLEK group (P<0.05). The following possible explanations for this difference were identified: i) the original donor may have exhibited corneal astigmatism, if the conditions mapping the pre-operative donor cornea lens have been selected; ii) a donor cornea may exhibit diseases that lead to corneal curvature and abnormal thickness (if the original cornea exhibited keratoconus angiogenesis, the rate of wound healing would be inconsistent); iii) inconsistencies may be present in the cut of the cornea, which may affect the the pre-operative preparations of the recipient, lead to a low intraocular pressure and result in the requirement of a speculum to open a sagging eye (leading to the cornea receptors being drilled into conical tissue); iv) the ring may deviate from the optical axis center (donor and/or recipient); v) the donor cornea may not match the recipient’s in shape and size; vi) the impact of the suture (the positioning of the sutures in the 4th, 2nd and 1st pin must be a straight line) or twisted sutures may result in needle spacing that is not uniform between the sutures; and vii) the surgeon may not have used the surgical keratometer to adjust the tightness of the sutures.

In addition to these points which aid in the prevention and treatment of astigmatism following surgery, the cornea and corneal lens measurements may be corrected using selective suture removal in the early post-operative period. Several authors have argued that wounds should be sutured with interrupted 12 needles and 10-0 nylon and that 11-0 nylon continuous sutures should be used with a 12-pin (19). Subsequent to recovery for 1 month, surgeons should remove 1 or 2 sutures every two weeks when the astigmatism is large, which will cause a more compact topography. Further study is required to determine an effective method to treat extreme astigmatism following suturing.

In summary, the results of the present study indicated that corneal graft rejections occur less often and are less severe following DLEK than following PK. In addition, the results indicate that post-operative corneal astigmatism following DLEK or PK differed in several ways. However, as visualized using H&E staining, the corneal shape appeared to be normal 3 months subsequent to the DLEK and PK surgeries. It is proposed that the topography of the cornea does not guarantee an improvement in visual acuity and stability. The present study did not examine the process of wound healing following DLEK surgery. The effect of the corneal topography on long-term vision should be studied further. If the patient maintains a relatively stable long-term corneal topography following surgery, the corneal interface in the recipient maintains optical transparency. If standardized surgical procedures, a proficiency in the surgical techniques and appropriate surgical instruments are available, it is feasible that DLEK may be an effective method to replace PK as a standard corneal transplantation method for treating BK.

## Figures and Tables

**Table I:** Comparison of corneal Astig values (D) at various time points following surgery (mean ± SD).

	Control group	BK 4 weeks	1 month after surgery	2 months after surgery	3 months after surgery	F	P-value
DLEK	1.08±0.25	2.43±0.24	2.62±0.21	2.29±0.34	2.07±0.19	827.84	0.00
PK	1.03±0.28	2.43±0.32	5.92±0.65	4.78±0.61	3.99±0.36	6071.82	0.00
t	-	-	110.68	82.242	43.666	-	-
P-value	-	-	0.00	0.00	0.00	-	-

Astig, astigmatism; SD, standard deviation; BK, bullous keratopathy; DLEK, deep lamellar endothelial keratoplasty; PK, penetrating keratoplasty.
